# 

**Published:** 1908-07

**Authors:** 


**CONTENTS—JULY, 1908.—VOL. XXIV, NO. 1.**

DEPARTMENT OF PUBLIC HEALTH—

The Texas Health Officers' Association.................. 1

Vital Statistics. By Dr. S. Burg, City Health Officer of San Antonio, Texas.................................. 10

Sanitation of Meat Markets. By Dr. B. T. Young, Assistant State Health Officer, Austin........................ 12

ORIGINAL ARTICLES—

Auto-Intoxication. By R. B. Sellers, M. D., Comanche, Texas ............................................ 15

A Tribute to the Medical Profession.................. 21

EDITORIAL DEPARTMENT—

The Tidal Wave of Sanitation.......................... 23

EDITORIALETS .......................................24-26

CORRESPONDENCE ....................................27-28

ABSTRACTS AND SELECTIONS...........................29-46

